# Role of NH_4_ ions in successive phase transitions of perovskite type (NH_4_)_2_ZnX_4_ (X = Cl, Br) by ^1^H MAS NMR and ^14^N NMR

**DOI:** 10.1039/c8ra01315b

**Published:** 2018-03-21

**Authors:** Ae Ran Lim

**Affiliations:** Analytical Laboratory of Advanced Ferroelectric Crystals, Jeonju University Jeonju 55069 Korea aeranlim@hanmail.net arlim@jj.ac.kr; Department of Science Education, Jeonju University Jeonju 55069 Korea

## Abstract

The ^1^H chemical shifts and the spin-lattice relaxation time, *T*_1ρ_, in the rotating frame of (NH_4_)_2_ZnX_4_ (X = Cl, Br) are observed in order to investigate local phenomena related to successive phase transitions. The temperature dependence of *T*_1ρ_ values for ^1^H showed a minimum, and the *T*_1ρ_ values for ^1^H appeared to be governed by tumbling molecular motions at high temperatures. In addition, ^14^N NMR spectra are studied in each phase of (NH_4_)_2_ZnX_4_ single crystals in the laboratory frame. The phase transition temperatures strongly affect the ^14^N number of symmetry related nitrogen centers within the unit cell. The ^1^H MAS NMR and ^14^N NMR results are discussed to elucidate the roles of NH_4_ ions during the phase transitions of (NH_4_)_2_ZnX_4_.

## Abstract 2

The ^1^H chemical shifts and the spin-lattice relaxation time, *T*_1ρ_, in the rotating frame of (NH_4_)_2_ZnX_4_ (X = Cl, Br) are observed in order to investigate local phenomena related to successive phase transitions.

## Graphical abstract

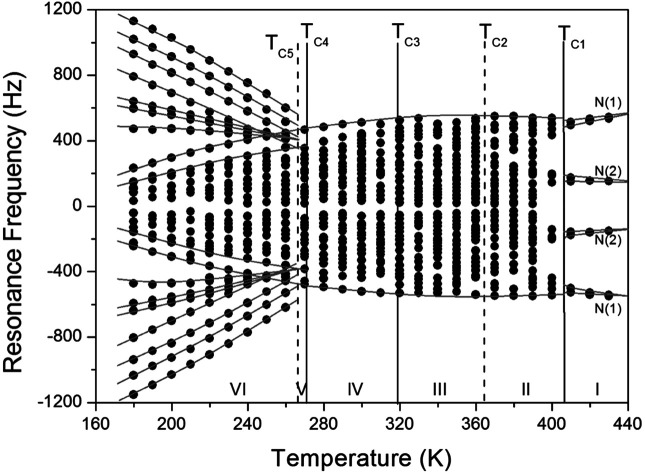

## 1. Introduction

Perovskite A_2_BX_4_ type (A = NH_4_, K, Rb, Cs; B = Zn, Co, Cu, Fe, Zn, Cd; X = Cl, Br) crystals have received a great deal of attention because of their nonlinear optical properties, and also because of the significant diversity of their structural phase transitions.^1–10^ The prototype of the crystal structures of this family is that of β-K_2_SO_4_, which consists of isolated BX_4_^2−^ tetrahedra and monovalent A^+^ cations placed in two inequivalent cavities. Ammonium tetrachlorozincate, (NH_4_)_2_ZnCl_4_, and ammonium tetrabromozincate, (NH_4_)_2_ZnBr_4_, belong to the family of crystals of the perovskite A_2_BX_4_ type and are known to undergo several phase transitions. Although the physical properties of (NH_4_)_2_ZnCl_4_ and (NH_4_)_2_ZnBr_4_ have been studied by several research groups, the structural geometry changes during the phase transitions of the two compounds have not been fully understood. Here, the phase transition temperatures and dynamics of the cations in (NH_4_)_2_ZnCl_4_ and (NH_4_)_2_ZnBr_4_ are important. The potential applications of these materials are strongly affected by the phase transitions and dynamics of the cations.^11^

(NH_4_)_2_ZnCl_4_ undergoes five phase transitions: those between phases I and II at 406 K (=*T*_C1_) and phases II and III at 364 K (=*T*_C2_) are well-known, and the successive phase transitions at 319 K (=*T*_C3_), 271 K (=*T*_C4_), and 266 K (=*T*_C5_) have also been reported;^12–14^ the phases involved in these transitions are denoted by VI, V, IV, III, II, and I in order of increasing temperature, as shown in Table 1. The structure of (NH_4_)_2_ZnCl_4_ in the normal phase, phase I (above 406 K), is orthorhombic with *a*_o_ = 9.274 Å, *b*_o_ = 12.620 Å, and *c*_o_ = 7.211 Å, and space group *Pnma*.^12^ Upon cooling, there is a phase transition at 406 K to an incommensurate phase that is stable down to 364 K.^15^ The structure in phase III between 364 K and 319 K is orthorhombic with *a* = *a*_o_, *b* = *b*_o_, *c* = 4*c*_o_, and the space group *Pn*2_1_*a*.^12,16,17^ The room temperature phase, phase IV, is antiferroelectric with a pseudo-orthorhombic monoclinic structure and space group *Pa*.^12^ The region between 271 K and 266 K is mixed phase.^14^ Below 266 K, the lattice is constant with an orthorhombic structure, where *a* = *a*_o_, *b* = *b*_o_, and *c* = 3*c*_o_.^12,17^

Phase transition temperatures, crystal structures, space groups, and lattice constants of (NH_4_)_2_ZnX_4_ (X = Cl, Br)

**Table:** 

(NH_4_)_2_ZnCl_4_						
Transition temperature	*T*_C5_ (=266K)	*T*_C4_ (=271 K)	*T*_C3_ (=319 K)	*T*_C2_ (=364 K)	*T*_C1_ (=406 K)	
Phase	VI	V	IV	III	II	I
Structure	Orthorhombic	Mixed phase	Monoclinic	Orthorhombic	Incommensurate	Orthorhombic
Space group	*Pna*2_1_		*Pa*	*Pn*2_1_*a*		*Pnma*
Lattice constant	*a* = *a*_o_		*a* = *a*_o_	*a* = *a*_o_		*a*_o_ = 9.274 Å
	*b* = *b*_o_		*b* = *b*_o_	*b* = *b*_o_		*b*_o_ = 12.620 Å
	*c* = 3*c*_o_		*c* = 4*c*_o_	*c* = 4*c*_o_		*c*_o_ = 7.211 Å
			*β* = 89.992°			
	*Z* = 12	*Z* = 12, 14, 16	*Z* = 16	*Z* = 16		*Z* = 4
Reference	12 and 17	14	12	12, 16 and 17	15	12

**Table:** 

(NH_4_)_2_ZnBr_4_				
Transition temperature	*T*_C3_ (=216 K)	*T*_C2_ (=395 K)	*T*_C1_ (=432 K)	
Phase	IV	III	II	I
Structure	Orthorhombic	Monoclinic	Incommensurate	Orthorhombic
Space group	*P*2_1_*cn*	*P2*_1_/*c*11		*Pmcn*
Lattice constant	*a* = *a*_o_	*a* = *a*_o_		*a*_o_ = 7.649 Å
	*b* = *b*_o_	*b* = *b*_o_		*b*_o_ = 13.353 Å
	*c* = 3*c*_o_	*c* = 4*c*_o_		*c*_o_ = 9.727 Å
		*β* = 90.00(3)°		
	*Z* = 12	*Z* = 16		*Z* = 4
Reference	22	22	20	21 and 22

**Fig. 1:**
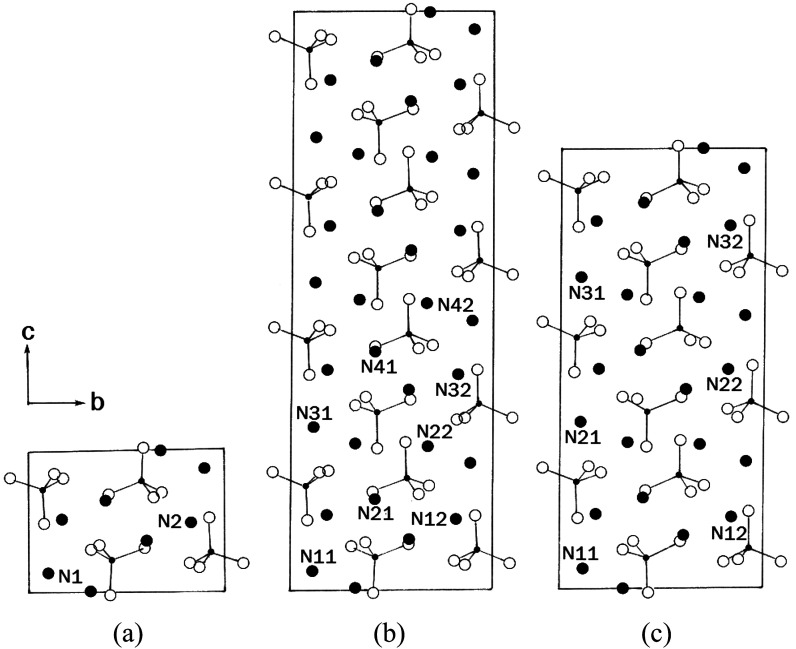
Projection of the structure along the *a*-axis in phases (a) I, (b) III, and (c) IV of (NH_4_)_2_ZnBr_4_ crystal.

On the other hand, the successive phase transitions of (NH_4_)_2_ZnBr_4_ have been reported at 216 K (=*T*_C3_), 395 K (=*T*_C2_), and 432 K (=*T*_C1_),^18–20^ as shown in Table 1; the phases involved in these transitions are represented by IV, III, II, and I in order of increasing temperature. The structures of (NH_4_)_2_ZnBr_4_ crystals in phases I, III, and IV are shown in Fig. 1. In phase I (above 432 K), the structure of (NH_4_)_2_ZnBr_4_ is orthorhombic with *a*_o_ = 7.649 Å, *b*_o_ = 13.353 Å, *c*_o_ = 9.727 Å, and space group *Pmcn*.^21,22^ Upon cooling, there is a phase transition at 432 K to an incommensurate phase II that is stable down to 395 K. The structure in phase III, between 395 K and 216 K, is monoclinic with *a* = *a*_o_, *b* = *b*_o_, *c* = 4*c*_o_, *β* = 90.00(3)°, and space group *P*2_1_/*c*11.^22^ The low-temperature phase IV is orthorhombic with *a* = *a*_o_, *b* = *b*_o_, *c* = 3*c*_o_, and space group *P*2_1_*cn*.^22^

The ^1^H spin-lattice relaxation times of (NH_4_)_2_ZnCl_4_ and (NH_4_)_2_ZnBr_4_ crystals have been obtained in the laboratory frame by Lim *et al.*^23–25^ and Ramesh *et al.*,^26^ respectively. The molecular dynamics and phase transitions of (NH_4_)_2_ZnCl_4_ single crystals were reported previously. There were two crystallographically inequivalent NH_4_ sites, namely NH_4_(1) and NH_4_(2), in the (NH_4_)_2_ZnCl_4_. The ^1^H spin-lattice relaxation time *T*_1_ in the laboratory frame was observed to vary continuously with temperature without jumps or changes. The ^1^H *T*_1_ passes through a minimum value near 220 K; the presence of this minimum was attributed to the reorientation of the NH_4_ groups. In addition, the ^14^N nuclear magnetic resonance (NMR) results in phase I of (NH_4_)_2_ZnCl_4_ were reported for the two inequivalent sites N(1) and N(2): the quadrupole coupling constant, *e*^2^*qQ*/*h*, and asymmetry parameter, *η*, were *e*^2^*qQ*/*h* = 105.5 kHz and *η* = 0.96 for N(1), and *e*^2^*qQ*/*h* = 48.2 kHz and *η* = 0.087 for N(2).^27^

The NMR method enables the study of a lattice's local properties, and is particularly useful in those cases that require information on the behavior of individual structural groups. Measurements of *T*_1ρ_ obtained by magic angle spinning (MAS) NMR in the rotating frame are advantageous in that they allow for probing of molecular motion in the kHz range, whereas *T*_1_ values obtained by state NMR in a laboratory frame reflect motion in the MHz range.^28^

The aim of this paper is to clarify the structural changes associated with the successive phase transitions in (NH_4_)_2_ZnX_4_ (X = Cl, Br). Detailed studies of the molecular motions are necessary in order to explain the mechanisms of the phase transitions of (NH_4_)_2_ZnX_4_. The temperature dependences of the MAS NMR spectra and the spin-lattice relaxation times, *T*_1ρ_, in the rotating frame for the ^1^H nuclei in (NH_4_)_2_ZnX_4_ were investigated using a pulsed NMR spectroscopy. In addition, the ^14^N NMR spectra in (NH_4_)_2_ZnX_4_ single crystals were obtained by static NMR in the laboratory frame, as a function of temperature. The ^1^H MAS NMR and ^14^N static NMR results were analyzed to elucidate the roles of NH_4_ ions during the phase transitions of (NH_4_)_2_ZnCl_4_ and (NH_4_)_2_ZnBr_4_. The *T*_1ρ_ values by ^1^H MAS NMR obtained here and the previously reported *T*_1_ values by ^1^H static NMR are compared. In addition, the information regarding the structural geometry of nitrogen environments in NH_4_^+^ is discussed as a function of temperature.

## 2. Experimental method

Single crystals of (NH_4_)_2_ZnCl_4_ and (NH_4_)_2_ZnBr_4_ were obtained by the slow evaporation of aqueous solutions with the appropriate molar ratios of NH_4_Cl and ZnCl_2_, and NH_4_Br and ZnBr_2_, respectively, at 298 K.^12,29,30^ These single crystals exhibited hexagonal shapes that were transparent and colorless.

^1^H MAS NMR spectra and the spin-lattice relaxation times, *T*_1ρ_, in the rotating frame in (NH_4_)_2_ZnCl_4_ and (NH_4_)_2_ZnBr_4_ were measured in a static magnetic field of 9.4 T and Larmor frequency of *ω*_0_/2π = 400.13 MHz, using a Bruker 400 MHz NMR spectroscopy at the Korea Basic Science Institute, Western Seoul Center. The chemical shifts were measured with respect to tetramethylsilane (TMS). Powder samples were placed inside a 4 mm cross-polarization (CP)/MAS probe, and the MAS rate was set to 5 kHz to minimize spinning sideband overlap. ^1^H *T*_1ρ_ values were determined using a π/2–*t* sequence by varying the duration of spin-locking pulses. The widths of the π/2 pulse used to measure the *T*_1ρ_ values of ^1^H in (NH_4_)_2_ZnCl_4_ and (NH_4_)_2_ZnBr_4_ were 4.35 μs and 3.7 μs, respectively, with the spin-locking field equaling 57.47 kHz and 67.56 kHz.

In addition, the ^14^N NMR spectra of the (NH_4_)_2_ZnCl_4_ and (NH_4_)_2_ZnBr_4_ single crystals in the laboratory frame were measured using a Unity INOVA 600 NMR spectroscopy at the Korea Basic Science Institute, Western Seoul Center. The static magnetic field was 14.1 T, and the Larmor frequency was set to *ω*_0_/2π = 43.342 MHz. The ^14^N NMR experiments were performed using a solid echo sequence of π/2–*t*–π/2–*t*. The widths of the π/2 pulse for ^14^N in (NH_4_)_2_ZnCl_4_ and (NH_4_)_2_ZnBr_4_ were 4 μs and 3.7 μs, respectively. The measurements of ^1^H MAS NMR in the rotating frame and ^14^N NMR in the laboratory frame were obtained over the temperature range of 180–430 K. Sample temperatures on MAS NMR and static NMR were held constant within ±0.5 K by controlling the helium gas flow and heater current.

## 3. Experimental results and discussion

### 3.1 Phase transition temperatures

**Fig. 2:**
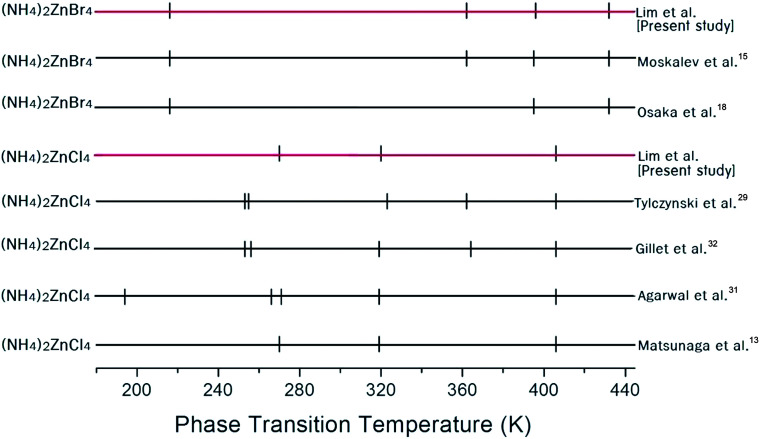
Phase transition temperatures (K) for (NH_4_)_2_ZnX_4_ (X = Cl, Br) reported by several groups.

The phase transition temperatures for (NH_4_)_2_ZnCl_4_ and (NH_4_)_2_ZnBr_4_ single crystals have not yet been accurately established, as shown in Fig. 2. Here, the small vertical bars were represented the phase transition temperatures reported by several groups. In the case of (NH_4_)_2_ZnCl_4_, this crystal exhibits three temperature dependence anomalies in the dielectric, thermal, and X-ray diffraction measurements at 270 K, 319 K, and 406 K, respectively, as reported by Matsunaga *et al.*^13^ Furthermore, anomalies characteristic of phase transitions reported by Agarwal *et al.*^31^ have been found in the Raman spectra investigations at 194 K, 266 K, 271 K, 319 K, and 406 K. According to Gillet *et al.*,^32^ the occurrence of phase transitions at 253 K, 256 K, 319 K, 364 K, and 406 K was also confirmed on the basis of the Brillouin investigation.^32^ In addition, thermal expansion changes at temperatures of 253 K, 255 K, 323 K, 362 K, and 406 K were reported by Tylczynski *et al.*^29^ In the case of (NH_4_)_2_ZnBr_4_, phase transition temperatures have been reported at 216 K, 395 K, and 432 K by Osaka *et al.*,^18^ and an additional phase transition at 365 K was reported by Moskalev *et al.*^15^ in their investigation of a (NH_4_)_2_ZnBr_4_ crystal using ^81^Br nuclear quadrupole resonance (NQR), differential thermal analysis (DTA), and dielectric measurements. Fig. 2 shows that the phase transition temperatures obtained by several experiments are inconsistent for (NH_4_)_2_ZnCl_4_ and (NH_4_)_2_ZnBr_4_, respectively.

**Fig. 3:**
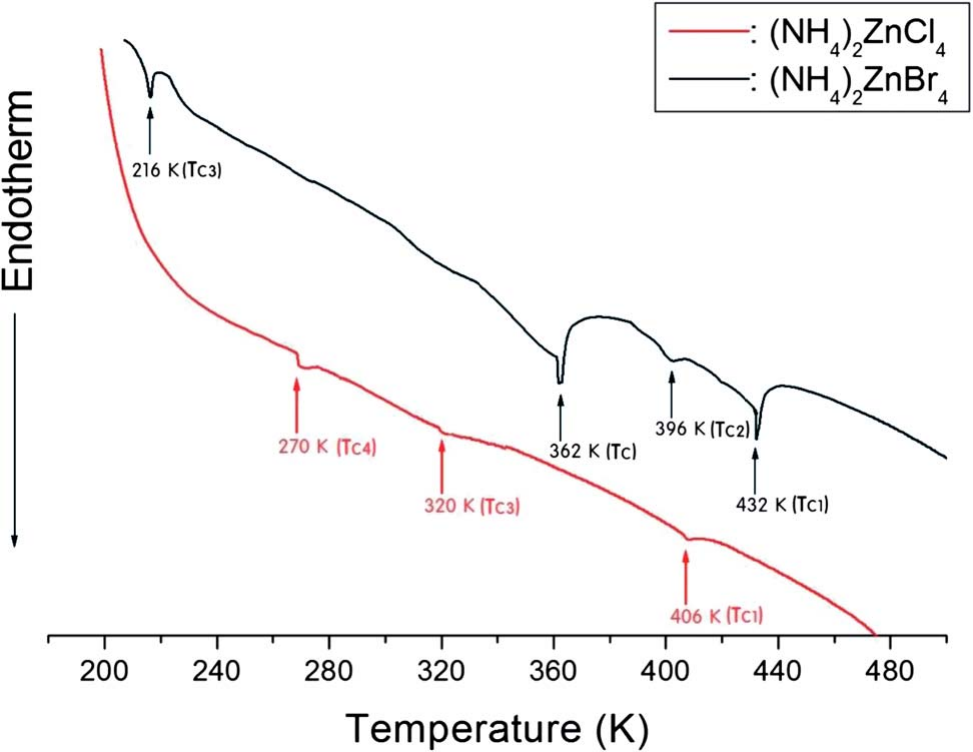
Differential scanning calorimetry (DSC) thermograms of (NH_4_)_2_ZnCl_4_ and (NH_4_)_2_ZnBr_4_ single crystals.

In order to determine the phase transition temperatures for the (NH_4_)_2_ZnCl_4_ and (NH_4_)_2_ZnBr_4_ single crystals obtained here, differential scanning calorimetry (DSC) measurements were taken with a DuPont 2010 DSC instrument at a heating rate of 10 °C min^−1^. The DSC measurements revealed three endothermic peaks at 270 K, 320 K, and 406 K for (NH_4_)_2_ZnCl_4_, and four endothermic peaks at 216 K, 362 K, 396 K, and 432 K for (NH_4_)_2_ZnBr_4_, as shown in Fig. 3. These endothermic peaks were related to the phase transitions, and the temperatures were consistent with those previously reported by Matsunaga *et al.*^13^ and Moskalev *et al.*^15^ The phase transition temperatures of (NH_4_)_2_ZnX_4_ may vary according to the conditions of crystal growth.

### 3.2 Molecular motion near phase transition temperatures from ^1^H MAS NMR

**Fig. 4:**
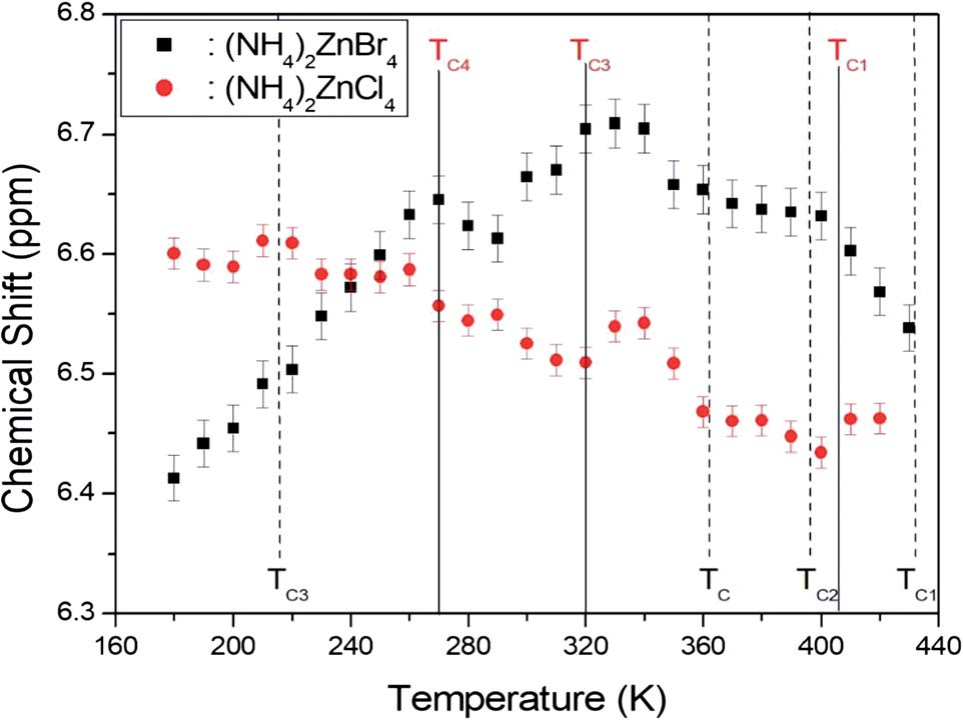
Chemical shifts of ^1^H MAS NMR spectra in (NH_4_)_2_ZnCl_4_ and (NH_4_)_2_ZnBr_4_ as a function of temperature ((NH_4_)_2_ZnCl_4_: solid lines, (NH_4_)_2_ZnBr_4_: dash lines).

The ^1^H MAS NMR spectra in (NH_4_)_2_ZnCl_4_ and (NH_4_)_2_ZnBr_4_ were measured as a function of temperature. At room temperature, (NH_4_)_2_ZnCl_4_ and (NH_4_)_2_ZnBr_4_ showed only one peak each, at chemical shifts of *δ* = 6.53 ppm and *δ* = 6.66 ppm, respectively, as shown in Fig. 4. The phase transition temperatures of (NH_4_)_2_ZnCl_4_ are denoted by solid lines, and those of (NH_4_)_2_ZnBr_4_ are denoted dash lines. The chemical shifts of the two materials did not change near the phase transition temperatures. The ^1^H chemical shifts of (NH_4_)_2_ZnCl_4_ decreased with increasing temperature, whereas the ^1^H chemical shift of (NH_4_)_2_ZnBr_4_ increased with increasing temperature. From the chemical shift for ^1^H, the proton environments in N–H⋯Cl bond and the proton environments in N–H⋯Br bond were very different. This difference of chemical shifts was possibly due to the difference between the electron structures of halogen ions.

**Fig. 5:**
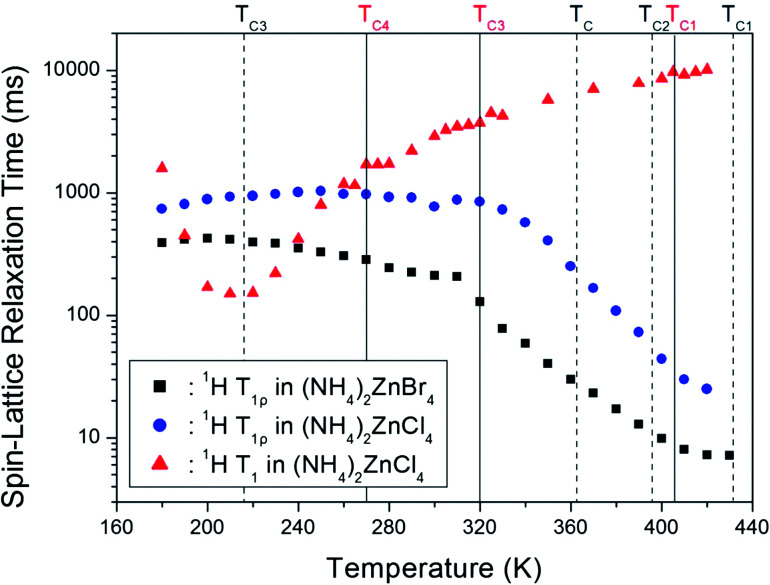
Temperature dependences of the spin-lattice relaxation time, *T*_1ρ_, in the rotating frame and the spin-lattice relaxation time, *T*_1_, in the laboratory frame of the ^1^H nuclei in (NH_4_)_2_ZnCl_4_ and (NH_4_)_2_ZnBr_4_ ((NH_4_)_2_ZnCl_4_: solid lines, (NH_4_)_2_ZnBr_4_: dash lines).

The decay traces for the ^1^H resonance line in (NH_4_)_2_ZnCl_4_ and (NH_4_)_2_ZnBr_4_ are represented by a single exponential function of *M*(*t*) = *M*(∞)exp(−*t*/*T*_1ρ_), where *M*(*t*) is the magnetization as a function of the spin-locking pulse duration *t*, and *M*(∞) is the total nuclear magnetization of ^1^H at thermal equilibrium.^14^ The decay traces for the ^1^H nuclei varied with the delay time, and these decay traces also varied depending upon the temperature. The decay traces fitted with the single exponential function for delay times. From the slopes of the decay traces, the ^1^H spin-lattice relaxation times, *T*_1ρ_, in the rotating frame for the (NH_4_)_2_ZnCl_4_ and (NH_4_)_2_ZnBr_4_ were obtained as a function of temperature, as shown in Fig. 5. The *T*_1ρ_ values of ^1^H were significantly different in the high-temperature and low-temperature regions. The significant difference in the *T*_1ρ_ values indicates that (NH_4_)_2_ZnCl_4_ and (NH_4_)_2_ZnBr_4_ are strongly affected, which considered to be mainly the result of molecular motions. The ^1^H *T*_1ρ_ data showed no evidence of a change near the phase transition temperatures. The trend of ^1^H *T*_1ρ_ in (NH_4_)_2_ZnCl_4_ resembled that of ^1^H *T*_1ρ_ in (NH_4_)_2_ZnBr_4_. The two *T*_1ρ_ series displayed similar trends, both decreasing quickly above 310 K. The variation of *T*_1ρ_ with temperature exhibited a shallow minimum of 7.2 ms at 430 K in the case of (NH_4_)_2_ZnBr_4_, indicating that distinct molecular motion is present. The *T*_1ρ_ minimum is considered to be clearly attributable to the tumbling motion of NH_4_^+^ ions. The *T*_1ρ_ values are related to the corresponding values of the rotational correlation time, *τ*_C_, which directly measures the rate of motion. The experimental *T*_1ρ_ value can be expressed in terms of *τ*_C_ using molecular motion, as suggested by the Bloembergen–Purcell–Pound (BPP) theory.^33^ The *T*_1ρ_ value in the rotating frame can also be expressed in terms of *τ*_C_ using molecular motion.^28,34^

1 1/*T*_1ρ_ = (*n*/20)(*γ*_H_*γ*_N_*ħ*/*r*_H–N_^3^)^2^[4*f*(*ω*_1_) + *f*(*ω*_H_ − *ω*_N_) + 3*f*(*ω*_N_) + 6*f*(*ω*_H_ + *ω*_N_) + 6*f*(*ω*_H_)]

Here:*f*(*ω*_1_) = *τ*_C_/[1 + *ω*_1_^2^*τ*_C_^2^]*f*(*ω*_H_ − *ω*_N_) = *τ*_C_/[1 + (*ω*_H_ − *ω*_N_)^2^*τ*_C_^2^]*f*(*ω*_N_) = *τ*_C_/[1 + *ω*_N_^2^*τ*_C_^2^]*f*(*ω*_H_ + *ω*_N_) = *τ*_C_/[1 + (*ω*_H_ + *ω*_N_)^2^*τ*_C_^2^]*f*(*ω*_H_) = *τ*_C_/[1 + *ω*_H_^2^*τ*_C_^2^]

In the equation, *γ*_H_ and *γ*_N_ are the gyromagnetic ratios for the ^1^H and ^14^N nuclei, respectively; *n* is the number of directly bound protons; *r*_H–N_ is the H–N internuclear distance; *ħ* is the reduced Planck constant; *ω*_H_ and *ω*_N_ are the Larmor frequencies of ^1^H and ^14^N, respectively; and *ω*_1_ is the spin-lock field frequency of 67.56 kHz. Here, the *f*(*ω*_1_) is non-zero, *i.e.*, the *τ*_C_ is much less than the Larmor frequencies, therefore all of the other terms is far smaller than the *f*(*ω*_1_) term. We analyzed our data by assuming that *T*_1ρ_ would show a minimum when *ω*_1_*τ*_C_ = 1, and that the relation between *T*_1ρ_ and the characteristic frequency of motion, 1/*τ*_C_, could be applied. The coefficient in eqn (1) can be determined because the *T*_1ρ_ curve displays a minimum and because the value of *τ*_C_ can be obtained from *ω*_1_*τ*_C_ = 1; thus, (*n*/20)(*γ*_H_*γ*_N_*ħ*/*r*_H–N_^3^)^2^ ≈ 4.66 × 10^6^ in the BPP formula. We were then able to calculate the correlation time *τ*_C_ as a function of temperature. The temperature dependence of *τ*_C_ follows a simple Arrhenius expression:^34,35^

2 *τ*_C_ = *τ*_o_ exp(−*E*_a_/*RT*),

where *τ*_o_ is a pre-exponential factor, *T* is the temperature, *R* is the gas constant, and *E*_a_ is an activation energy. Thus, the slope of the linear portion of a semi-logarithmic plot should yield *E*_a_. The value of *E*_a_ for the tumbling motion can be obtained from a plot of log *τ*_C_*versus* 1000/*T*. We obtained *E*_a_ = 36.69 ± 0.66 kJ mol^−1^ for the tumbling motion of ^1^H in (NH_4_)_2_ZnBr_4_ at high temperature. This value is very similar to that for ^1^H in (NH_4_)_2_ZnCl_4_, and is the same within the error range. And, the *E*_a_ for (NH_4_)_2_ZnCl_4_ and (NH_4_)_2_ZnBr_4_ at low temperature is 0.32 ± 0.30 kJ mol^−1^ and 2.25 ± 0.37 kJ mol^−1^, respectively.

On the other hand, the ^1^H spin-lattice relaxation time *T*_1_ in the laboratory frame in (NH_4_)_2_ZnCl_4_ previously reported was obtained as a function of temperature, as shown in Fig. 5.^23–25^ In the high temperature region, *T*_1_ increases monotonically with temperature, and *T*_1_ was continuous at the phase transition temperatures. However, the *T*_1_ undergoes a change in slope near *T*_C4_. ^1^H *T*_1_ passes through a minimum value in the vicinity of 220 K, and the presence of this minimum was attributed to the effects of molecular motion. The relaxation process from the ^1^H *T*_1_ curve was affected by molecular motion, as described by the BPP theory.^33^ The activation energy in the low and high temperatures was reported 29.95 ± 0.85 kJ mol^−1^ and 10.99 ± 0.37 kJ mol^−1^, respectively.

### 3.3 Structural changes near phase transition temperatures from ^14^N NMR

**Fig. 6:**
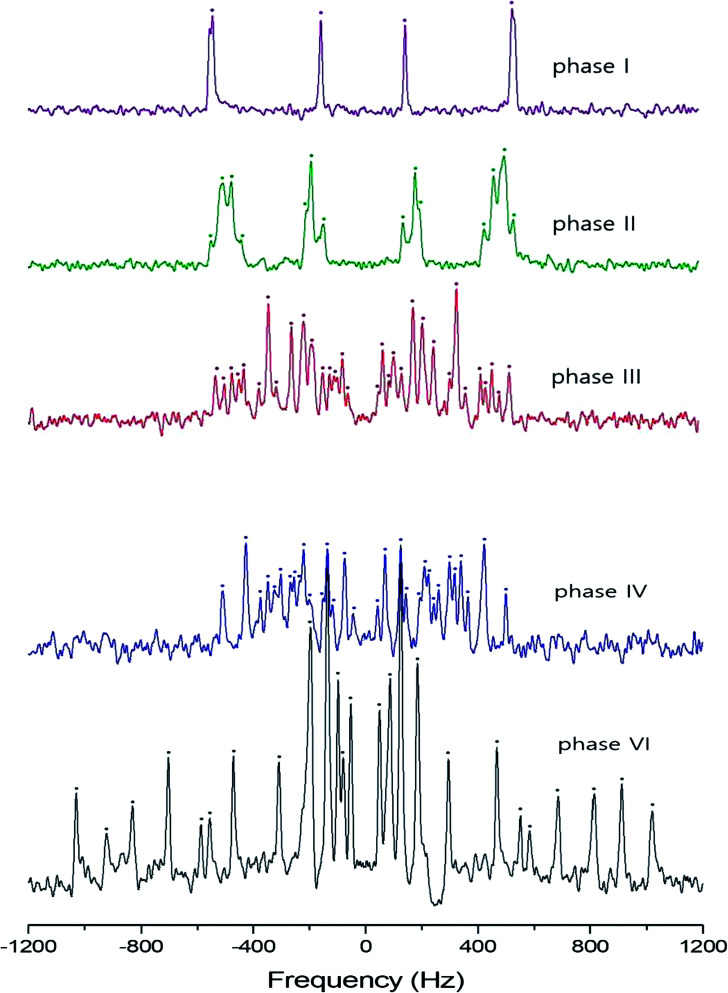
^14^N NMR spectra in (NH_4_)_2_ZnCl_4_ single crystal at phase I, II, III, IV, and VI.

In order to investigate local phenomena related to successive phase transitions, the NMR spectra of ^14^N (*I* = 1) was obtained as a function of temperature using static NMR at a Larmor frequency of *ω*_0_/2π = 43.342 MHz. ^14^N (*I* = 1) NMR is a sensitive method for probing local structural properties in each phase. The ^14^N NMR spectra consisted of pairs of lines at frequencies corresponding to the transitions Δ*m* = ±1 ↔ Δ*m* = 0. The crystal was oriented such that the magnetic field was aligned with the crystallographic *c*-axis. Temperature-dependent changes in the ^14^N resonance frequency are generally attributed to changes in the structural geometry, indicating a change in the quadrupole coupling constant of the ^14^N nuclei. The ^14^N NMR spectra at phase I, II, III, IV, and VI in (NH_4_)_2_ZnCl_4_ crystals were plotted in Fig. 6. Here, the ^14^N peaks positions were denoted by close circles. Two resonance lines were expected because of the quadrupole interaction of the ^14^N nucleus. However, many resonance lines were observed, and they were much narrower in line width.

**Fig. 7:**
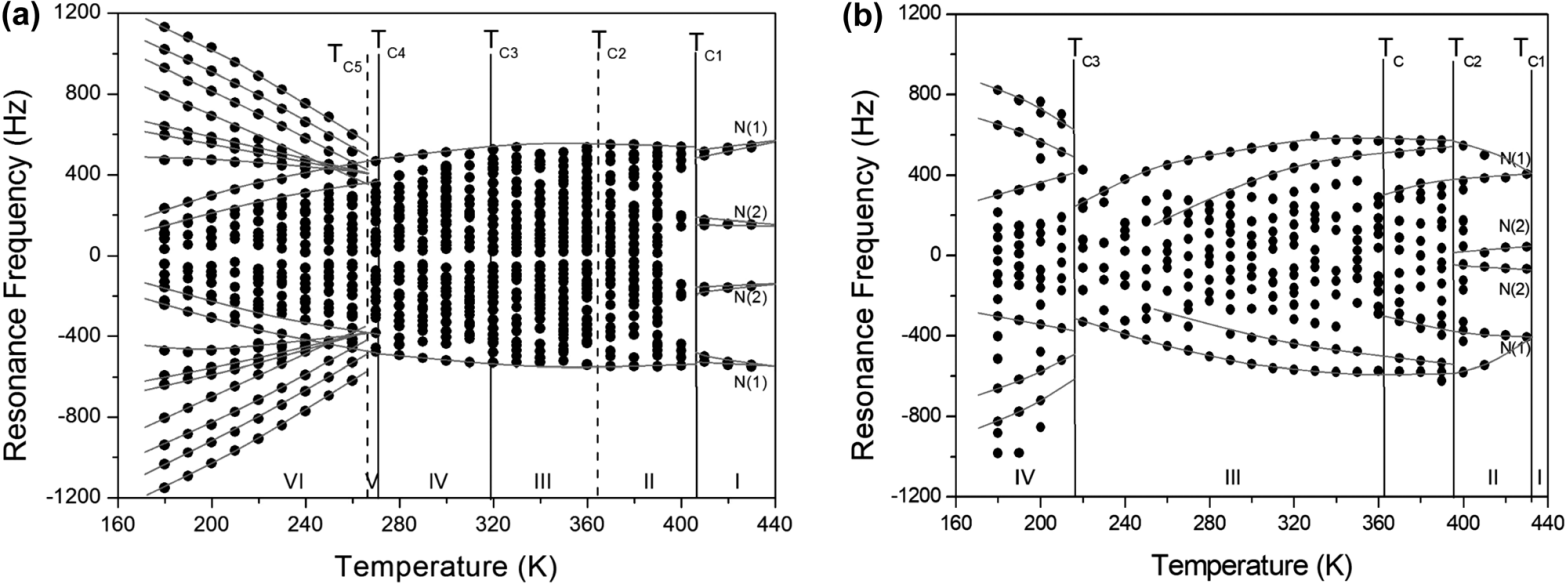
(a) Resonance frequency of ^14^N NMR spectra in (NH_4_)_2_ZnCl_4_ single crystal as a function of temperature. (b) Resonance frequency of ^14^N NMR spectra in (NH_4_)_2_ZnBr_4_ single crystal as a function of temperature.

The resonance frequencies of ^14^N signals in (NH_4_)_2_ZnCl_4_ and (NH_4_)_2_ZnBr_4_ single crystals are respectively plotted in Fig. 7(a) and (b) as a function of temperature. In the case of (NH_4_)_2_ZnCl_4_, the phase transitions occurring at *T*_C1_, *T*_C3_, and *T*_C4_ were observed from our DSC results, whereas those at *T*_C2_ and *T*_C5_ were not observed. Therefore, *T*_C1_, *T*_C3_, and *T*_C4_ are denoted by solid lines, and *T*_C2_ and *T*_C5_ are denoted dotted lines in Fig. 7(a). The resonance frequencies near *T*_C1_, *T*_C4_, and *T*_C5_ changed, whereas those near *T*_C2_ and *T*_C3_ did not change. In phase I, each unit cell contains four formula units, and there are also two different kinds of ^14^N nuclei, termed N(1) and N(2). Therefore, the ^14^N NMR spectra exhibited eight resonance lines in four pairs. Here, the two inequivalent sites N(1) and N(2) are distinguished by the quadrupole coupling constant previously reported: *e*^2^*qQ*/*h* = 105.5 kHz and *η* = 0.96 for N(1), and *e*^2^*qQ*/*h* = 48.2 kHz and *η* = 0.087 for N(2).^27^ Additional lines in phases II, III, and IV were obtained, although they exhibited very small intensities compared with phase I. In phases III and IV, the unit cell is quadrupoled along the *c*-direction of phase I. The unit cell of phases III and IV contains 16 formula units, and thus 32 resonance lines of 16 pairs are expected. According to the crystallography results shown in Fig. 1(b), eight atoms N(11), N(21), N(31), and N(41) are surrounded by five Cl atoms, while the other atoms N(12), N(22), N(32), and N(42), which are located between the layers created by the ZnCl_4_ tetrahedra, are surrounded by eight Cl atoms. From the NMR spectra results of 16 pairs of ^14^N, the approximately 32 resonance lines in phases III and IV were measured, as shown in Fig. 7(a). The 32 resonance lines from the 16 pairs of ^14^N in the NH_4_ ion were consistent with the previously reported crystallography structure.^12,16,17^ In addition, phase VI, below *T*_C5_, contains *Z* = 12 formula units, N(11), N(12), N(21), N(22), N(31), and N(32), as shown in Fig. 1(c). Therefore, approximately 24 resonance lines in 12 pairs were obtained. In these results, the splitting of the ^14^N resonance lines for seven of the pairs slightly decreased with increasing temperature, whereas those of the ^14^N resonance lines for the other five pairs slightly increased with increasing temperature. On the other hand, the resonance frequencies in phases I, II, III, and IV for the case of (NH_4_)_2_ZnBr_4_ are shown in Fig. 7(b). The ^14^N NMR spectra from (NH_4_)_2_ZnBr_4_ could not be easily observed in detail because of their very low intensity. However, the resonance frequencies near *T*_C2_ and *T*_C3_ changed discontinuously.

As mentioned above, in the case of (NH_4_)_2_ZnCl_4_, four pairs of lines ascribed to N(1) and N(2) nuclei appeared in phase I, above 406 K. Because the frequency distributions in phase II discontinuously emerged from these high-temperature lines, the notations N(1) and N(2) were not retained. Based on this temperature dependence, the assignment of the resonance lines in phases III and IV are also not denoted. The resonance frequency from phase V cannot be distinguished because of the much narrower temperature range. The resonance frequencies in phase VI, below *T*_C4_, changed discontinuously from those of phases V and IV. The changes in the resonance lines near *T*_C4_ for (NH_4_)_2_ZnCl_4_ and *T*_C3_ for (NH_4_)_2_ZnBr_4_ also indicated a phase transition where the new phase exhibited orthorhombic symmetry, which equates to a higher degree of symmetry compared with monoclinic symmetry. Abrupt changes in the resonance frequencies for ^14^N near the phase transition temperatures are generally attributed to structural phase transitions.

## 4. Conclusion

Data on structural geometries near successive phase transition temperatures of (NH_4_)_2_ZnX_4_ (X = Cl, Br) were obtained by ^1^H MAS NMR and ^14^N NMR as a function of temperature. We studied the molecular motions in (NH_4_)_2_ZnX_4_, based on the ^1^H chemical shifts and spin-lattice relaxation time, *T*_1ρ_, in the rotating frame. The ^1^H chemical shifts near the phase transition temperatures for the two materials did not show any drastic change, and this result might be related to proton ordering near the phase transition temperatures. From the ^1^H *T*_1ρ_ results, the activation energies for the tumbling motion of ^1^H had very similar values, and the tumbling motion of NH_4_^+^ ions occurred within the high-temperature range.

We compared the ^1^H MAS NMR in the rotating frame measured here and the previously reported ^1^H static NMR results in the laboratory frame^23–25^ for (NH_4_)_2_ZnCl_4_. The trends in *T*_1ρ_ values for ^1^H in (NH_4_)_2_ZnCl_4_ are different from the trends in the *T*_1_ values. The molecular motion by *T*_1ρ_ in the rotating frame was dominant at high temperature, whereas that by *T*_1_ in the laboratory frame was dominant at low temperature. The activation energy values extracted from *T*_1ρ_ and *T*_1_ measurements are different for the molecular motions in the kHz and MHz ranges.

The ^14^N NMR spectra exhibited a sudden shift in the ^14^N peak positions and number of peaks at the phase transition temperatures. The electric field gradient (EFG) tensors at the N sites varied, reflecting the changing atomic configurations around the ^14^N nuclei. This is because the phase transition temperature strongly affect the ^14^N number of symmetry related nitrogen centers within the unit cell. Therefore, ^14^N NMR provides insight into changes in crystal symmetry and cation reorientation rates induced by heating and phase transitions.

The two crystals have different phase transition temperatures, but seemingly similar phase transition mechanisms. Although (NH_4_)_2_ZnX_4_ has different bond lengths in the Zn–X (X = Cl, Br) structure, and different X atomic radii, the different halide ions (X = Cl, Br) do not appear to significantly influence the ^1^H relaxation time.

## Conflicts of interest

There are no conflicts to declare.

## Supplementary Material

## References

[cit1] Griset C., Head S., Alices J., Starykh O. A. (2011). Phys. Rev. B.

[cit2] Ono T., Tanaka H., Shirata Y., Kindo A., Ishikawa F., Kolomiyets O., Mitamura H., Goto T., Nakano N., Fortune N. A., Hannahs S. T., Yoshida Y., Takano Y. (2011). J. Phys.: Conf. Ser..

[cit3] Vachon M., Koutroulakis G., Mitrovic V. F., Ma O., Marston J. B., Reyes A. P., Kuhns P., Coldea R., Tylczynski Z. (2011). New J. Phys..

[cit4] Foyevtsova K., Opahle I., Ahang Y.-Z., Jeschke H. O., Valenti R. (2011). Phys. Rev. B.

[cit5] Coletta T., Zhitomirsky M. E., Mila F. (2013). Phys. Rev. B.

[cit6] Zvyagin S. A., Kamenskyi D., Ozerov M., Wosnitza J., Ikeda M., Fujita T., Hagiwara M., Smirnov A. I., Soldatov T. A., Ya Shapiro A., Krzystek J., Hu R., Ryu H., Petrovic C., Zhitomirsky M. E. (2014). Phys. Rev. B.

[cit7] Yamada A. (2014). Phys. Rev. B.

[cit8] Merino J. (2014). Phys. Rev. B.

[cit9] Zvyagin S. A., Ozerov M., Kamenskyi D., Wosnitza J., Krzystek J., Yoshizawa D., Hagiwara W., Hu R., Ryu H., Petrovic C., Zhitomirsky M. E. (2015). New J. Phys..

[cit10] Schmidt B., Thalmeier P. (2015). New J. Phys..

[cit11] Kubicki D. J., Prochowicz D., Hofstetter A., Pechy P., Zakeeruddin S. M., Gratzel M., Emsley L. (2017). J. Am. Chem. Soc..

[cit12] Matsunaga H. (1982). J. Phys. Soc. Jpn..

[cit13] Matsunaga H., Nakamura E. (1981). J. Phys. Soc. Jpn..

[cit14] Sata T., Osaka T., Makita Y. (1984). J. Phys. Soc. Jpn..

[cit15] Moskalev A. K., Belobrova I. A., Zherebtsova L. I., Aleksandrova I. P. (1982). Phys. Status Solidi A.

[cit16] Mikhail I. (1980). Acta Crystallogr., Sect. B: Struct. Crystallogr. Cryst. Chem..

[cit17] Matsunaga H., Ithoh K., Nakamura E. (1982). Acta Crystallogr., Sect. B: Struct. Crystallogr. Cryst. Chem..

[cit18] Osaka T., Komukae M., Makita Y. (1982). J. Phys. Soc. Jpn..

[cit19] Koningsveld H. V. (1983). Acta Crystallogr., Sect. C: Cryst. Struct. Commun..

[cit20] Sato T., Osaka T., Makita Y. (1983). J. Phys. Soc. Jpn..

[cit21] Asker W. J., Scaife D. E., Watts J. A. (1972). Aust. J. Chem..

[cit22] Shigematsu H., Kasano H., Mashiyama H. (1993). J. Phys. Soc. Jpn..

[cit23] Lim A. R. (2011). Solid State Commun..

[cit24] Lim A. R., Lim K.-Y. (2013). J. Solid State Chem..

[cit25] Lim A. R. (2012). Chem. Phys..

[cit26] Ramesh K. P., Devaraj N., Vijayaraghavan D., Ramakrishna J. (1992). Phase Transitions.

[cit27] Michel D., Muller B., Petersson J., Trampert A., Walisch R. (1991). Phys. Rev. B.

[cit28] Koenig J. L. , Spectroscopy of Polymers, Elsevier, New York, 1999

[cit29] Tylczynski Z., Piskunowicz P. (1990). Phys. Status Solidi A.

[cit30] Strivastava J. P., Kulshreshtha A. (1991). J. Opt. Soc. Am. B.

[cit31] Agarwal A., Patel M. B., Pal M., Bist H. D. (1984). Spectrochim. Acta, Part A.

[cit32] Gillet A. M., Luspin Y., Hauret G. (1987). Solid State Commun..

[cit33] Bloembergen N., Purcell E. M., Pound R. V. (1948). Phys. Rev..

[cit34] Lim A. R. (2016). AIP Adv..

[cit35] Abragam A. , The Principles of Nuclear Magnetism, Oxford University Press, 1961

